# Burden and care time for dementia caregivers in the LIVE@Home.Path trial

**DOI:** 10.1002/alz.14622

**Published:** 2025-03-05

**Authors:** Line Iden Berge, Renira Corinne Angeles, Marie Hidle Gedde, Stein Erik Fæø, Janne Mannseth, Maarja Vislapuu, Natalie Genevieve Søyland Puaschitz, Eirin Hillestad, Dag Aarsland, Wilco Peter Achterberg, Heather Allore, Clive Ballard, Fan Li, Geir Selbæk, Ipsit Vihang Vahia, Bettina Sandgate Husebo

**Affiliations:** ^1^ Center for Elderly and Nursing Home Medicine Department of Global Public Health and Primary Care Faculty of Medicine University of Bergen Bergen Norway; ^2^ NKS Olaviken Gerontopsychiatric Hospital Erdal Norway; ^3^ NORCE Norwegian Research Centre Bergen Norway; ^4^ Haraldsplass Deaconess Hospital Bergen Norway; ^5^ Faculty of Health Studies Department of Nursing VID Specialized University Bergen Norway; ^6^ Section for Epidemiology and Medical Statistic Department of Global Public Health and Primary Care Faculty of Medicine University of Bergen Bergen Norway; ^7^ Western Norway University of Applied Sciences Bergen Norway; ^8^ The Dignity Centre Bergen Norway; ^9^ Institute of Psychiatry Psychology and Neuroscience King's College London UK; ^10^ Department of Public Health and Primary Care Center for Medicine for Older People Leiden University Medical Center Leiden the Netherlands; ^11^ Department of Internal Medicine School of Medicine Yale University New Haven Connecticut USA; ^12^ Department of Biostatistics Yale School of Public Health New Haven Connecticut USA; ^13^ University of Exeter Medical School Exeter UK; ^14^ Center for Methods in Implementation and Prevention Science Yale School of Public Health New Haven Connecticut USA; ^15^ Norwegian National Advisory Unit on Ageing and Health Vestfold Hospital Trust Tønsberg Norway; ^16^ Institute of Clinical Medicine Faculty of Medicine University of Oslo Oslo Norway; ^17^ Geriatric Department Oslo University Hospital Oslo Norway; ^18^ McLean Hospital Belmont Massachusetts USA; ^19^ Harvard Medical School Boston Massachusetts USA; ^20^ Neuro‐SysMed Center Department of Global Public Health and Primary Care University of Bergen Bergen Norway

**Keywords:** care coordination, caregiver burden, dementia, resource use, stepped wedge randomized controlled trial

## Abstract

**INTRODUCTION:**

We investigated the effectiveness of the multicomponent learning, innovation, volunteer support, empowerment (LIVE) intervention on caregiver burden and care time in dyads of home‐dwelling people with dementia and caregivers.

**METHOD:**

A 24 month, multicenter, stepped‐wedge trial, randomized dyads to receive the 6‐month LIVE intervention by municipal coordinators (May 2019 to December 2021). Primary outcomes were caregiver burden assessed by Relative Stress Scale (RSS) and informal care time spent on personal activities assessed by Resource Utilization in Dementia Personal Activities of Daily Living (RUD‐PADL). Analyses used an intention‐to‐treat.

**RESULTS:**

Two hundred eighty dyads were enrolled. Caregivers during the intervention period reported lower levels of RSS of 0.7 points (standard deviation [SD]: 0.8) compared to the caregivers in the control period. Caregivers during the intervention period reported more time spent on PADL of 11.7 hours/month (SD: 8.7) compared to caregivers during the control period; both were not statistically significant (*P* > 0.05).

**DISCUSSION:**

The LIVE intervention did not reduce caregiver burden or care time.

**TRIAL REGISTRATION:**

ClinicalTrials.gov NCT04043364.

**Highlights:**

- Two hundred eighty persons with dementia and caregivers were included in a stepped wedge randomized controlled trial.
- We used the learning, innovation, volunteer support, empowerment (LIVE) intervention.
- The LIVE intervention did not reduce caregiver burden or informal care time.
- The LIVE intervention improved the caregiver's clinical global impression of change.
- Positive change was most pronounced for coordinator personalized support.

## 1 BACKGROUND

The global old‐age dependency ratio will rise over the next decades due to fewer births and lower mortality rates.^1^, ^2^ The increased life expectancy may accompany more years lived at home with complex chronic conditions and multimorbidity, including dementia.^3^ Worldwide, 57 million people have dementia, expected to reach 153 million by 2050.^4^ Dementia is the single most expensive disease in Norway, and close to 80% of the formal costs are attributed to nursing home care.^5^ Yet, in Norway, 70% of people with dementia are living at home, supported by the political and economic incentives.^6^ Moreover, there is a trend of deinstitutionalization in Western European countries, which shifts the caring burden to the informal caregivers, who already experience high care burden.^1^

Care for people with dementia is time consuming and associated with the experiences of stress and anxiety when the demands of caregiving exceed available resources. To mitigate this caregiver burden and to support families at home, several randomized controlled trials have investigated non‐pharmacological interventions targeting caregivers to people with dementia. The most promising of these are multicomponent interventions, that is, interventions with at least two components.^7^ For instance, the REACH II randomized controlled trial by Berwig et al. provided informal caregivers with social support, cognitive behavioral techniques, and education over 5 months and found a positive effect on caregiver burden over the 3 months of follow‐up.^8^ Another German trial, the cluster randomized DelpHi study by Thyrian et al. included 643 home‐dwelling persons with dementia and implemented a collaborative model of care with a trained dementia care manager over 6 months.^9^ This intervention reduced the caregiver burden and also improved behavioral and psychological symptoms at 12 month follow‐up.^9^ Both studies highlighted the need for proper implementation of the intervention and sufficient follow‐up. A limitation with these parallel arm trials is that the multicomponent interventions are difficult to blind and consequently more prone to biased self‐reported outcomes and risk of loss of motivation and drop‐out in the control group.^9^ Additionally, due to cultural differences in policy and organization, stakeholder involvement is crucial in the development of the intervention content. As such, there is a need for locally developed multicomponent intervention trials with rigorous methods to increase the benefits and generalizability of such implementation programs.^6^

The LIVE@Home.Path is a stepped‐wedge randomized controlled trial that aimed to support people with dementia and their informal caregivers to live safely and independently at home through increased literacy about dementia and related services.^10^ LIVE is the acronym for the multicomponent intervention encompassing Learning, Innovation, Volunteer support, and Empowerment defined as closer collaboration with the general practitioner. All components were developed by user representatives and implemented by trained municipal coordinators. We adopted a stepped‐wedge design that provides all participants with the intervention over three intervention periods.^11^ When the SARS CoV‐2 pandemic hit Norway on March 12, 2020, quarantine was imposed; thus, we adapted the protocol including delivery of the intervention, data collection, and statistical analysis plan in accordance with the pandemic context.^12^

In this study, we investigate our primary hypothesis that the LIVE intervention may reduce (1) the caregiver burden through increased literacy and support by the municipal coordinator and (2) number of care hours provided by caregivers to people with dementia residing at home, as some of the time‐consuming caring tasks were to be supported by assistive technology and volunteer services. Secondarily, we tested whether the caregivers would report improvements in quality of life, symptoms of depression, and a positive impression of change over the trial. To summarize, our primary aim is to assess the effectiveness of the LIVE intervention on the primary outcomes of caregiver burden and informal care time for personal activities of daily living (PADL). Our secondary aims are to investigate the impact of the LIVE intervention on informal care time for instrumental activities and supervision, in addition to informal caregivers’ quality of life, symptoms of depression, and their awareness of the Clinical Global Impression of Change (CGIC).

## 2 METHODS

### 2.1 Design

**FIGURE 1 alz14622-fig-0001:**
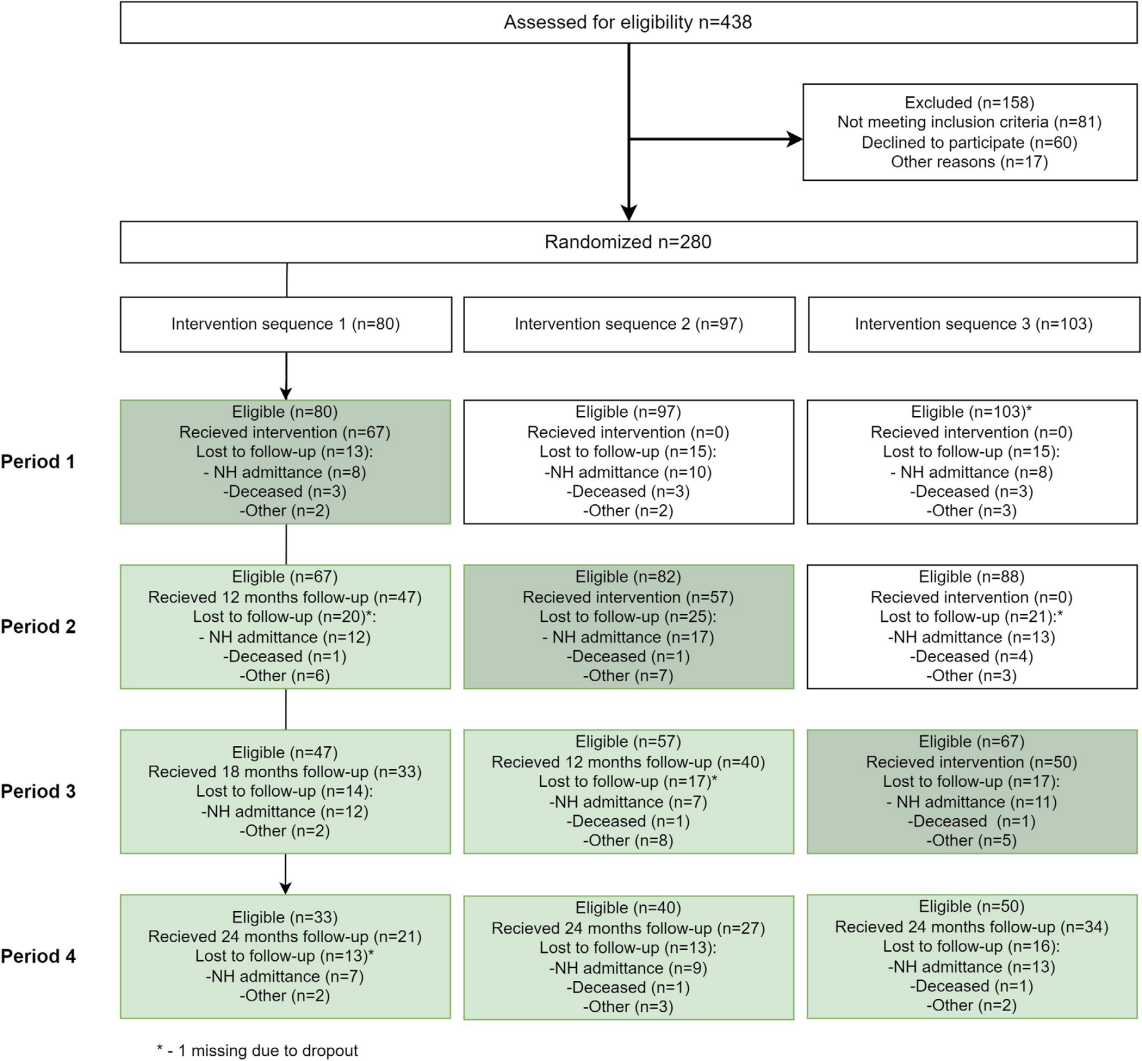
Flow chart of the LIVE@Home.Path stepped wedge randomized controlled trial. NH, nursing home.

From May 2021, we conducted a stepped‐wedge randomized controlled trial in three municipalities across Southern Norway (Bergen, Bærum, and Kristiansand). A stepped‐wedge trial is a one‐way cross‐over trial in which units are randomized to different intervention sequences with different transition points (i.e., steps).^11^ In our trial, the unit of randomization was the dyad, consisting of a person with dementia and their informal caregiver. All dyads were recruited before randomization, exposed to both the control and the 6‐month intervention period, and assessed every subsequent 6 months (Figure 1). This yielded baseline and four periods of 6 months’ duration at discrete times during the 24 month trial. The trial included a total of three steps, and each step allocated approximately one third of the recruited dyads from each municipality from the control to the intervention period. The intervention was delivered over three 6‐month periods between months 0 and 18, while the period from 18 to 24 months was follow‐up of all participants remaining in the trial. Each dyad contributed with time in the control condition, intervention condition (6 months), and follow‐up condition; the amount of time in the control and follow‐up condition was determined by randomization (Figure 1). Data were collected at the dyads’ homes by study personnel (clinical research team and municipal staff from the home‐care services) blinded to allocation status; the personnel conducted separate interviews with both the person with dementia and the caregiver. The trial ended upon completion in December 2021. The LIVE@Home.Path was approved by the Regional Committee for Medical and Health Research Ethics, North Norway (2019/385). The protocol for the trial, including rationale and method development, has been reported in detail.^10^ Trial registration is at ClinicalTrials.gov: NCT04043364.

**RESEARCH IN CONTEXT**

1. **Systematic review**: We performed a systematic review (PROSPERO CRD42021226388) identifying 29 studies evaluating factors that could reduce informal care hours in dementia caregivers. We were inspired by the Norwegian Dementia Plan's recommendations combining actions such as educational support, technology, and volunteerism to support home‐dwelling persons with dementia and caregivers. The efficacy of these interventions has not yet been explored in the real‐world community setting.
2. **Interpretation**: The learning, innovation, volunteer support, empowerment (LIVE) intervention led to no significant change in caregiver burden and informal care time. The caregivers reported a positive clinical global impression of change regarding all the LIVE components, most pronounced for personalized support provided by the coordinators.
3. **Future directions**: This finding may support clinicians and stakeholders designing future health‐care services for people with dementia and caregivers. Future studies should evaluate the cost effectiveness of the implementation of a trained coordinator tailoring services in municipal dementia care.

### 2.2 Participants

We recruited dyads from geriatric and gerontopsychiatric outpatient clinics, municipal memory teams, and the general media without financial incentives. Potential participants were identified by collaborating staff in the services. Home‐dwelling persons with dementia and their informal caregivers were eligible for inclusion[Fig alz14622-fig-0001] as a dyad if the person with dementia was (1) aged ≥ 65 years, (2) self‐reported being diagnosed with dementia, (3) had Mini‐Mental State Examination (MMSE)^13^ score between 15 and 25, and (4) a Functional Assessment Staging Tool (FAST)^14^ score between 4 and 7. Participants were eligible regardless of dementia etiology and presence of other conditions. Exclusion criteria were participation in another ongoing trial and expected survival < 4 weeks. Informal caregivers were eligible for inclusion if they had minimum 1 hour/week regular face‐to‐face contact with the person with dementia. Dyads were included by study personnel between May 2019 and September 2019. Prior to inclusion, spoken and written informed consent was obtained in direct conversation with all the included caregivers and persons with dementia, if capable of providing consent for participation. If not, the informal caregiver or legal advocate provided consent based on their determination of whether the person with dementia would have agreed to participate when he or she was able to make this decision.

### 2.3 Randomization and masking

We selected a stepped‐wedge design with randomization performed at the dyad level. This design was chosen because it ensures that each dyad will receive the LIVE intervention during the study, and fewer coordinators were needed as they could deliver the intervention in a staggered fashion. All dyads started from the usual care condition and were then switched to receive the intervention until all dyads were exposed under the intervention condition. The study statistician used a computerized random number generator to determine the randomization order in which dyads were allocated to the three intervention sequences within each of the three municipalities, that is, stratified randomization by municipality. The sequence allocation was concealed from the research assistants, researchers, and other study personnel who conducted enrollment and data collection. The sequence allocation was also concealed for the dyads until contacted by their coordinator at the start of the intervention period. The coordinators were informed about allocation status when their designated dyads entered the intervention period, yet allocation was concealed from outcome assessors.

### 2.4 Procedures

In the intervention condition, based on the recommendations in the National Dementia Plan by the Norwegian Ministry of Health and Care Services,^6^ our team developed the content of the LIVE intervention (Table 1) together with stakeholders. This process was tailored to meet the standards of the “development–evaluation–implementation” framework, an international approach for complex interventions by the UK Medical Research Council,^15^ and we performed a feasibility study to evaluate the users’ perceptions on care support, including the coordinator function, assistive technology, and volunteer support.^16^ The coordinators were nurses, assistant nurses, and occupational therapists experienced in dementia care already working in the home‐based services of the designated municipalities. The implementation of the LIVE intervention was conducted in two steps.^10^ The first part included activities by the research team to empower the coordinators to standardize the implementation. This included written material (tutorial, manual, checklist), a 2‐day implementation seminar before each intervention period, a 1‐day midway evaluation, and bi‐weekly phone calls by the research team during the intervention period. The second part included activities performed by the coordinator to implement the intervention to the dyads. At the start of the intervention period, the coordinator visited each dyad at home and introduced them to the specific components of the LIVE intervention (Table 1). It encompassed learning programs provided by the municipality and hospital trusts on dementia‐related topics (the disease, legal rights, safety, economy, and coping), implementation of assistive technology available in the designated municipalities (passive and active sensors, video communication), introduction to the local volunteer services, and initiation of contact with the general practitioner to safeguard medication review and advanced care planning. After 3 months, the coordinators provided a follow‐up visit, in addition to monthly phone follow‐up or home visits during the 6‐month intervention period. During the follow‐up condition, the dyads were offered the option to contact the coordinator if they had additional needs.

**TABLE 1:** The multicomponent intervention in the LIVE@Home.Path trial.

	Learning	Innovation	Volunteer support	Empowerment
Content	Learning programs on dementia ‐ Etiology, symptoms, and disease course ‐ Legal rights ‐ Safety ‐ Economy ‐ Coping	Assess the need for, evaluate the usefulness of, and inform about relevant assistive technology ‐ Passive sensors ‐ Active sensors and tracking devices ‐ Video communication	Explore attitudes toward volunteer services and initiate contact with non‐profit organizations	Establish contact with the regular general practitioner to initiate: ‐ Advanced care planning ‐ Medication review
Provider	Municipality hospital trust	Coordinator	The Red Cross Norwegian Association of Public Health	General practitioner
Participants	‐ PwD ‐ Caregiver ‐ Coordinator	‐ PwD ‐ Caregiver ‐ Coordinator	‐ PwD ‐ Caregiver ‐ Coordinator ‐ Volunteers from non‐profit organizations matched by volunteer managers	‐ PwD ‐ Caregiver ‐ Coordinator ‐ PwD's regular general practitioner

*Note*: Each component of the intervention was implemented by a municipal coordinator. The coordinators were nurses, learning disability nurses, and occupational therapists experienced in dementia care already working in the home‐based services in the municipalities. During the intervention period, each coordinator served 5 to 7 dyads in addition to other municipal tasks not affiliated with the trial.

Abbreviation: PwD: person with dementia.

In Norway, dementia and elderly care is primarily a municipal responsibility, and municipal health services strive to offer the “lowest effective level of care.” This is represented by the home health care service, which in addition to home nursing includes assistive home care, delivery of meals, and day‐care centers. The recipients can have a maximum of 80 hours/week with home health care service while the medical follow‐up is provided by general practitioners. We designated this regular home health service “care as usual” and it served as the control condition.

### 2.5 Outcomes

The primary outcome measures were caregiver burden assessed with the Relative Stress Scale (RSS)^17^ and informal resource use for PADLs (mobility, toileting, hygiene, and eating) assessed by the Resource Utilization in Dementia Personal Activities of Daily Living (RUD‐PADL; range 0–480 hours/month).^18^ RSS is a self‐report instrument covering three dimensions of “emotional distress,” “social distress,” and “negative feelings” attributed to caring (range 0–60 points), with higher scores indicating higher burden; RSS of 23 is the established cut‐off in the literature suggesting high risk of psychiatric morbidity.^17^ RUD assesses total care hours that informal caregivers provided in the past 30 days in three dimensions of care: personal ADL (RUD‐PADL); instrumental ADL (RUD‐Instrumental Activities of Daily Living [IADL], for example, medication, preparing meals, household chores, range 0–480 hours/month); and supervision to prevent adverse events, such as falls or wandering outdoors (RUD‐supervision, range 0–720 hours/month).^18^ The sum score of RUD‐PADL, RUD‐IADL, and RUD‐supervision cannot exceed 720 hours/month. Decrease in hours of informal care, as well as lower score of RSS are interpreted as a reduction in caregiver burden. Secondary outcomes were RUD‐IADL and RUD‐supervision, in addition to European Quality of Life‐5 Dimensions‐5 Levels (EQ‐5D‐5L),^19^ Geriatric Depression Scale (GDS),^20^ and CGIC,^21^ all reported by the caregivers. EQ‐5D‐5L evaluates generic self‐reported health‐related quality of life in relation to resource use covering mobility, self‐care, activities, pain/discomfort, and anxiety/depression.^19^ Scores are converted to a single summary index number (range 0–1), with higher scores indicating higher health‐related quality of life.^19^ The GDS is a screening instrument for depressive symptoms in older adults (range 0–30), with higher scores indicating higher symptom load.^20^ The CGIC scale quantifies and tracks patient progress and intervention response in clinical trials on an 11‐point scale from −5 to 5, in which 0 represents no clinical change, while a positive change is regarded as a beneficial change.^21^ In our trial, CGIC was assessed by the caregivers in the interviews with the study personnel. It was adapted to measure the caregiver's perception of meaningful change related to the overall situation and change related to each of the four LIVE components (learning, innovation, volunteering, and empowerment) and the coordinator. A similar formulation has been applied in nursing homes.^22^ Data on RSS, RUD, EQ‐5D‐5L, and GDS were assessed during the interviews with the caregiver every 6 months, in total five times during the 24‐month trial. As CGIC assesses change over time, data on CGIC were obtained during interviews with the caregiver at 6, 12, 18, and 24 months.

### 2.6 Statistical analysis

The original sample size was calculated to detect a difference of 7hours/week for the primary outcome RUD‐PADL.^10^, ^18^ Based on the literature, we assumed that the mean number of hours of informal care is 46 hours/week with a standard deviation (SD) of 20 hours/week.^23^ With 80% power and a significance level of 5%, the required sample size was estimated to equal 260 dyads. To allow for ≈ 20% loss to follow‐up, a total of 315 dyads, equaling 105 per municipality, were needed. This original sample size calculation did not consider the staggered intervention delivery and is therefore likely conservative.^24^ To reflect the nature of the staggered intervention delivery, we performed additional power analyses under an individually randomized stepped‐wedge trial with three intervention sequences and five periods. As a conservative calculation, we assume 80 dyads per sequence, so that the total sample size is no larger than the original calculation and allows for a balanced assignment per sequence. To detect the effect size of 7hours/week in RUD‐PADL, we assume a within‐individual correlation of 0.1 over the 24‐month trial (for repeated outcome measures) and the calculation suggested that the power would be 95.0%. The study power remains > 94.8% when we vary the within‐individual correlation between 0 and 1 (Figure S1A in supporting information). For RSS, we assume the effect size of 3.5 units, SD of 11,^17^ and a within‐individual correlation of 0.5 over the 24‐month trial. Assuming 80 dyads per sequence leads to 98.1% power to detect this effect size in RSS. Sensitivity analyses indicate that power remains > 90% when we vary the within‐individual correlation between 0 and 1 (Figure S1B).

We used linear mixed‐effects models to estimate the intervention effect on the primary and secondary outcomes, with a random effect to account for the repeated observations at the dyad level (person with dementia or caregiver level, depending on the level of outcome measurement). Data from all enrolled dyads were included in these intention‐to‐treat analyses with 24‐month follow‐up.

First, intervention effects on primary and secondary outcomes were modeled with a time‐invariant intervention effect,^25^, ^26^ according to the primary analysis plan. The model includes fixed effects for time to adjust for secular trend, fixed effects for municipality (stratification variable), and a single intervention effect parameter according to the treatment status for the dyads. To ensure that the analysis is robust to outcomes missing at random, baseline covariates (sexes for the person with dementia and the caregiver, age of caregiver, caregivers’ relation to the person with dementia, etiology of dementia, presence of pain disorders, and PADL total score) which may be related to missingness were included. In a post hoc analysis exploring the relative contribution of the LIVE components on the CGIC, we analyzed CGIC for each of the four LIVE components (learning, innovation, volunteer support, empowerment) and the coordinator.

Second, due to the Norwegian SARS CoV‐2 pandemic, we performed an additional linear mixed‐effects model with time‐dependent intervention effect to analyze how the intervention was affected by calendar time of the lockdown and the potential for delayed or sustained effect. Based on the principles from Li et al.,^25^ we modeled the intervention effect patterns over the different time periods and intervention sequences as follows: (1) a pre‐lockdown intervention effect, (2) a post‐lockdown intervention effect, and (3) a post‐lockdown follow‐up intervention effect (Table S1 in supporting information). All analyses were performed with Stata (version 17.0).[Table alz14622-tbl-0002]

The LIVE@Home.Path did not have a data safety monitoring board, as the trial was deemed low risk. Study investigators monitored adverse events during the trial, including falls, wandering outdoors, and death.

### 2.7 Changes to the trial protocol

On March 12, 2020, the Norwegian government implemented a quarantine to limit the spread of SARS CoV‐2 virus. Restrictions encompassed stay‐at‐home regulations, which implied that health‐care services were scaled down and restricted to those most in need. In the LIVE@Home.Path, the SARS CoV‐2 pandemic halted the trial protocol 8 to 12 weeks from March 2020 immediately after the end of the first intervention period and second data collection.^12^ Due to the shifting restrictions over the next 18 months and temporary shutdown of some of the LIVE components (e.g., learning courses and volunteer services), we had to postpone and later modify the implementation of the intervention to the dyads in sequences 2 and 3. We also allowed for follow‐up by phone by the coordinator while physical distancing was recommended as part of the SARS CoV‐2 policies.^12^ Given the disruptions of the SARS CoV‐2 pandemic, we were unable to analyze the trial in a joint model context as proposed in the protocol,^10^ as the dates of drop‐out were not recorded for the randomized dyads. The changes in design were approved by the regional ethical committee (REK: 10861).

## 3 RESULTS

Between May 25 and August 31, 2019, a total of 438 dyads were screened for participation, of which 280 were enrolled in the trial. Eighty dyads were randomized to intervention sequence 1 and of these, 67 received the intervention; corresponding numbers of dyads in the second and third intervention sequences were 97/57 and 103/50, respectively. The flow chart of the participating dyads, time periods, reasons for drop‐out, and drop‐out by intervention sequences are presented in Figure 1. A total of 15.4% dyads dropped out of the trial in period 1, 27.8% in period 2, 28.0% in period 3, and 34.1% in period 4 (follow‐up); the main reason for drop out was nursing home admission. Table 2 presents baseline characteristics of the persons with dementia and caregivers, stratified by intervention sequence. At baseline, mean age of the person with dementia was 82.3 years (SD: 6.8), 63% were female, and 49% resided with the reporting caregiver. Among the persons with dementia, 54.6% reported that they had dementia of unspecified etiology and 45% were in good health, while mean MMSE was 20.6 points (SD: 3.7) and mean FAST score 4.3 points (SD: 1.4). The mean age of the caregiver was 66.0 years (SD: 12.3), 65% were female, and 50.7% were the person with dementia's child. A total of 52.0% of the caregivers reported that they contributed between 81% and 100% of the care for the person with dementia. All persons with dementia and all caregivers were ethnic White Norwegians. At baseline, the caregivers reported an average of 30.7 (SD: 34.0) hours/month spent on PADL, 37.0 (48.6) hours/month on IADL, and 94.4 (SD: 129.4) hours/month on supervising. Mean RSS was 15.9 (SD: 10.3), 24.3% had RSS ≥ 23 points, EQ‐5D‐5L was 0.8 (SD: 0.1), and GDS was 5.0 (SD: 4.6). A total of 35.5% of the dyads reported adverse events at baseline, 31.6% at 6 months, 30.6% at 12 months, 26.6% at 18 months, and 28.7% at 24 months; most of the adverse events were falls (data not shown). We found no adverse events that were unexpected, and none judged to be related to study participation (data not shown).

**TABLE 2 alz14622-tbl-0002:** Baseline characteristics of persons with dementia and caregivers in the LIVE@Home.Path trial (*N* = 280).

	Total sample (*n* = 280)	Intervention Group 1 (*n* = 80)	Intervention Group 2 (*n* = 97)	Intervention Group 3 (*n* = 103)
**Person with dementia**				
Age in years, mean (SD)	82.3 (6.8)	83.9 (6.9)	81.0 (7.3)	81.8 (6.5)
Sex, female (%)	62.8	70.9	61.8	7.4
Living situation				
Living alone (%)	48.9	55.3	40.5	52.0
Co‐residing with the reporting caregiver (%)	49.2	42.3	57.5	46.9
Co‐residing with someone else than the reporting caregiver (%)	1.9	2.5	2.0	1.0
Dementia etiology				
Alzheimer's disease (%)	36.5	34.2	39.2	35.6
Vascular dementia (%)	3.9	1.3	5.1	5.0
Lewy body dementia/frontotemporal dementia (%)	1.5	0.0	3.1	0.9
Dementia in other diseases classified elsewhere (%)	2.9	2.5	2.1	4.0
Unspecified dementia (%)	55.2	62.0	50.5	54.5
MMSE (SD) (range 0–30)	20.6 (3.7)	20.9 (3.4)	20.8 (3·7)	20.2 (4.0)
Functional assessment staging tool mean (SD) (range 1–7)	4.3 (1.4)	4.5 (1.4)	4.2 (1.4)	4.2 (1.3)
General medical health rating scale				
Poor health (%)	2.5	1.2	6.2	.
Fair health (%)	33.2	43.5	29.1	28.8
Good health (%)	46.8	39.7	47.9	51.5
Excellent health (%)	17.3	15.3	16.7	19.7
PADL mean (SD) (range: 6–30)	10.3 (3.3)	11.0 (3.7)	10.0 (3.3)	10.0 (3.0)
Instrumental activities of daily living mean (SD) (range: 8–31)	19.9 (6.1)	20.3 (6.1)	19.4 (6.3)	20.1 (5.8)
Neuropsychiatric inventory‐12 mean (SD) (range 0–144)	16.6 (15.6)	17.7 (15.1)	16.0 (15.8)	16.4 (16.0)
**Caregiver**				
Age in years, mean (SD)	66.0 (12.3)	65.9 (13.0)	66.9 (12.2)	65.2 (12.0)
Sex, female (%)	64.7	64.1	63.	66.4
Kinship to the person with dementia				
Spouse (%)	43.3	38.5	48.9	41.6
Child (%)	51.6	56.4	45.8	54.5
Other (%)	5.1	5.1	5.3	3.9
Caregiver's contribution to care				
81–100% (%)	51.7	50.0	53.2	51.5
61–80% (%)	17.7	14.1	22.3	16.2
41–60% (%)	16.7	19.2	12.8	18.2
1–40% (%)	13.9	16.7	11.7	19.5
Dyads receiving home nursing during the last 30 days (%)	53.9	50.0	75.0	49.0
Dyads accessing day care center during the last 30 days (%)	29.6	23.7	37.1	27.1
Dyads receiving home help the last 30 days (%)	34.6	36.2	28.8	50.0
RUD				
RUD‐PADL hours/month	30.7 (34.0)	27.5 (39.3)	36.6 (36.6)	27.5 (26.0)
RUD‐IADL hours/month	37.0 (48.6)	31.0 (37.3)	45.2 (49.2)	34.2 (55.3)
RUD‐Supervision hours/months	94.4 (129.4)	63.5 (89.9)	108.3 (150.0)	110.4 (136.2)
RSS mean (SD) (range: 0–60)	15.9 (10.3)	15.9 (9.5)	16.6 (11.1)	15.3 (10.2)
RSS ≥23 points (%)	24.3	19.1	27.6	22.3
EQ‐5D‐5L mean (SD) (range: 0–1)	0.8 (0.1)	0.8 (0.1)	0.8 (0.1)	0.9 (0.1)
GSD mean (SD) (range 0–30)	5.0 (4.6)	5.5 (4.3)	4.9 (5.1)	4.7 (5.2)

*Note*: Baseline characteristics of the persons with dementia and caregivers (*N* = 280) in the LIVE@Home.Path, overall and stratified according to intervention group. A lower score on the MMSE indicates greater cognitive impairment. A higher score on Functional Assessment Staging Tool indicates poorer functioning. A higher score on PADL and IADL Scales indicates higher dependency. A higher score on Neuropsychiatric Inventory indicates frequent and severe neuropsychiatric symptoms. RUD, number of hours/months provided by caregivers on PADL, IADL, and Supervision. A higher score on the RSS indicates a higher caregiver burden. A higher score on EQ‐5D‐5L indicates high satisfaction in life. A higher score on the GSD indicates more severe depressive symptoms. Missing ranged from 2% (age, sex of persons with dementia and their caregivers) to 80% (RUD‐PADL: 78.3%, RUD‐IADL: 55.7%, RUD‐Supervision: 80.2%).

Abbreviations: EQ‐5D‐5L, European Quality of Life‐5 Dimensions‐5 Levels; GSD, Geriatric Depression Scale; IADL, instrumental activities of daily living; MMSE, Mini‐Mental State Examination; PADL, personal activities of daily living; RSS, Relative Stress Scale; RUD, resource use in dementia; SD, standard deviation.

### 3.1 Time‐invariant intervention effect analysis on primary outcome RSS and RUD‐PADL

**FIGURE 2:**
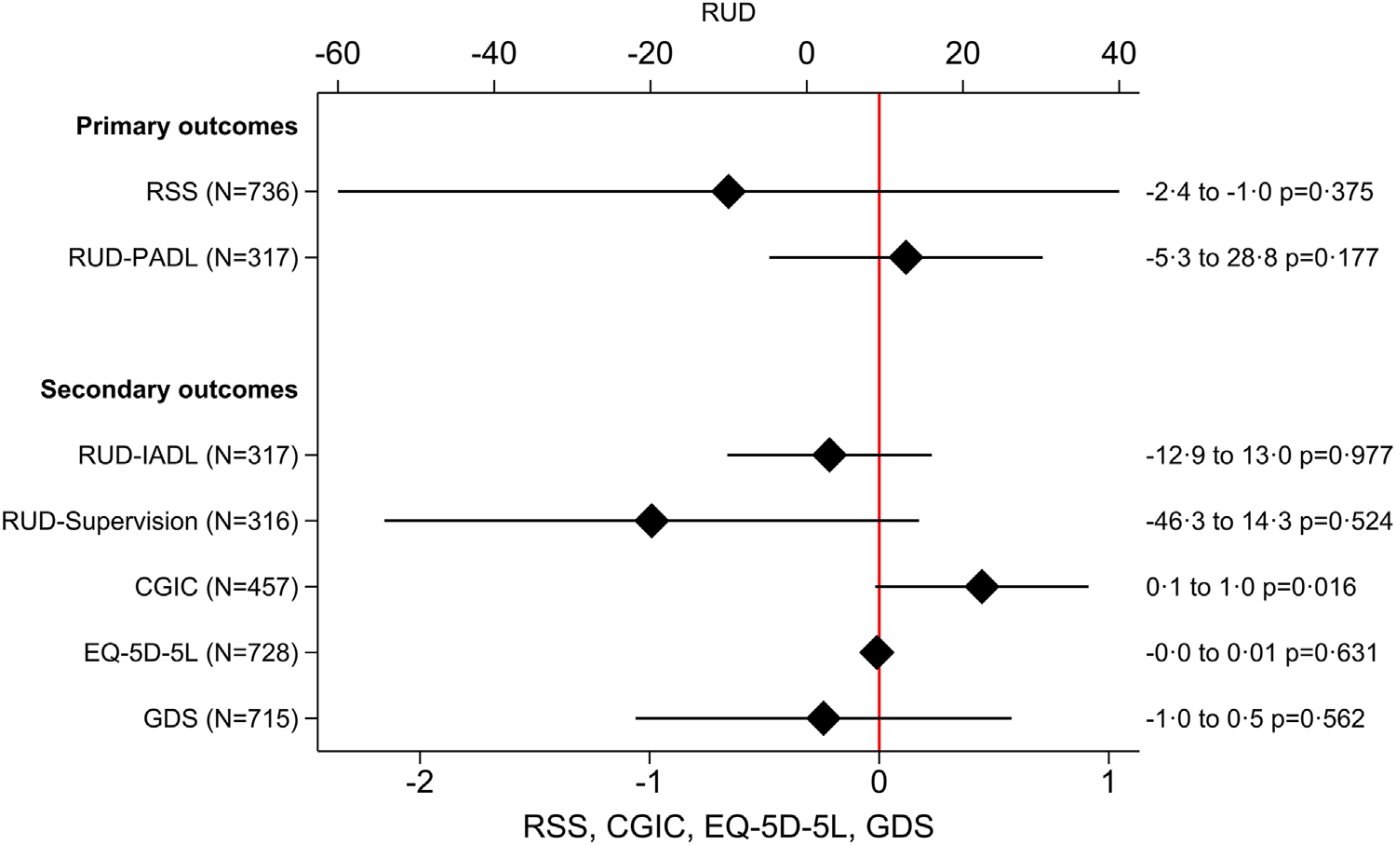
Mixed effect regression with time‐invariant intervention effect – primary and secondary outcomes. All models are adjusted for baseline covariates: sexes of the person with dementia and caregiver, caregiver age, relation to the person with dementia, dementia etiology, presence of pain disorders, and personal activities of daily living (total score). N represents the number of observations analyzed in the regression model. CGIC, Clinical Global Impression of Change; EQ‐5D‐5L, European Quality of Life‐5 Dimensions‐5 Levels; GSD, Geriatric Depression Scale; IADL, instrumental activities of daily living; PADL, personal activities of daily living; RSS, Relative Stress Scale; RUD, resource utilization in dementia.

After all intervention periods and the follow‐up period (24 months), caregivers during the intervention period reported on average lower levels of RSS of 0.7 points (SD: 0.8) compared to the caregivers in the control period (Figure 2). Moreover, caregivers during the intervention period reported on average more time spent on PADL of 11.7 hours/month (SD: 8.7) compared to the dyads during the control period (Figure 2). The effects of the intervention on the two primary outcomes were not statistically significant (*P* > 0.05).

### 3.2 Time‐invariant intervention effect analysis on secondary outcomes

The LIVE intervention led to no significant change in RUD‐IADL, RUD‐supervision, EQ‐5D‐5L, and GDS (Figure 2). After the intervention period, caregivers reported 0.5 points higher CGIC (95% confidence interval [CI]: 0.1–1.0) compared to the control period (Figure 2). A post hoc analysis on CGIC overall (the four LIVE components and the coordinator) and CGIC for each of the four LIVE components and the coordinator, showed that caregivers reported a notable increase in CGIC for all outcomes after the active intervention period (Figure S2 in supporting information). This effect was most pronounced for the coordinator with a positive change of 1.7 (95% CI: 1.4–2.0).

### 3.3 Time‐dependent intervention effect on primary outcome RSS and RUD‐PADL

**FIGURE 3:**
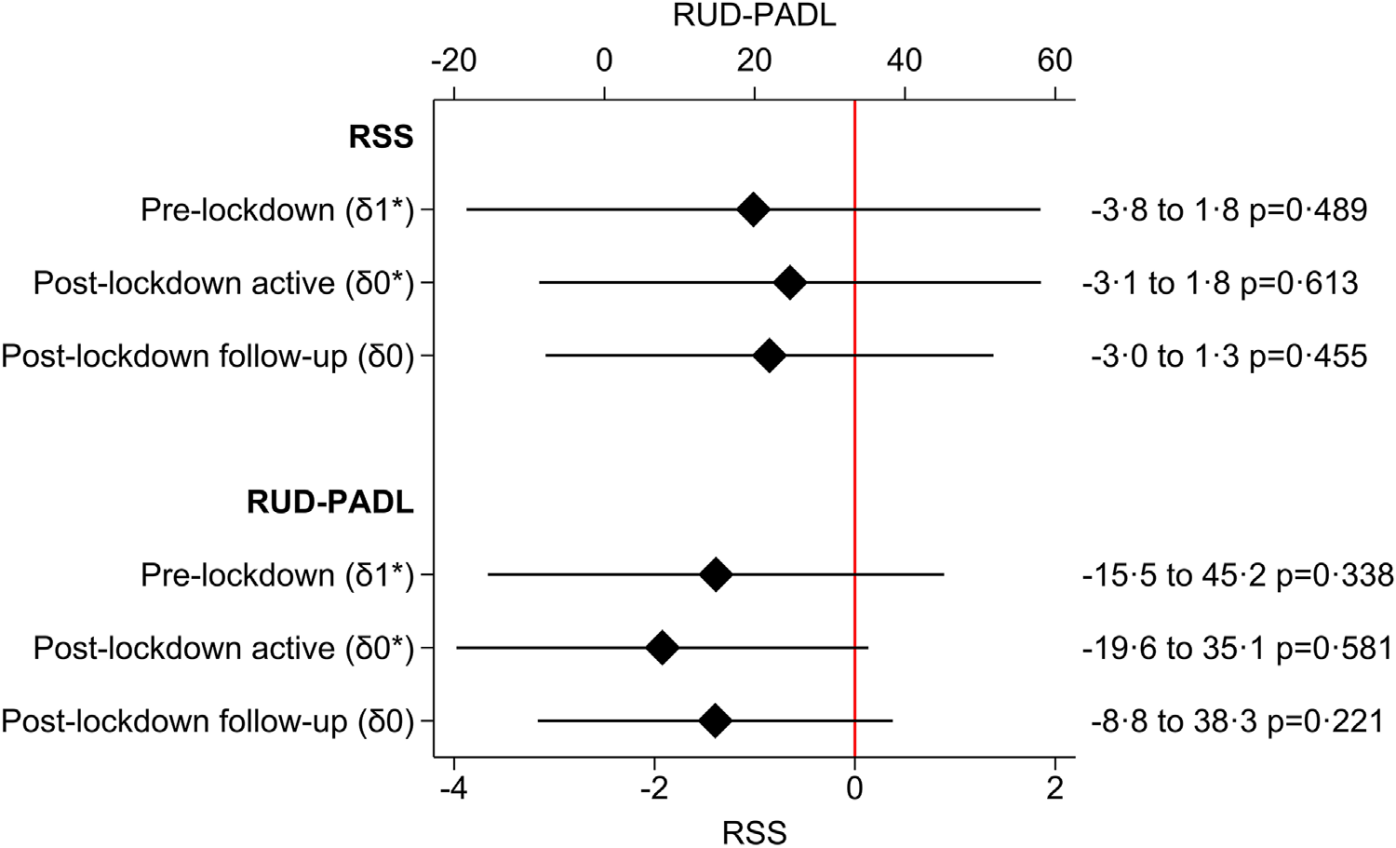
Mixed effect regression with time‐dependent intervention effect – primary outcomes The deltas (δ) constitute the intervention effect, and their subscript defines heterogenous effects where δ_1_^*^ = 1 if follow‐up post‐lockdown treatment effect δ_0_^*^ = 1 if post‐lockdown, and δ_0_ = 1 if pre‐lockdown intervention effect. All models are adjusted for baseline covariates: sexes of the person with dementia and caregiver, caregiver age, relation to person with dementia, dementia etiology, pain disorders and personal activities of daily living (total score). PADL, personal activities of daily living; RSS, Relative Stress Scale; RUD, resource utilization in dementia.

The mixed effect regression model with time‐dependent intervention effects did not show any statistically significantly delayed or sustained intervention effects on the primary outcomes (Figure 3). Based on recommendations from the Consolidated Standards of Reporting Trials extensions to stepped‐wedge designs,^27^ we report the within‐individual correlation estimates from all models in Table S2 in supporting information.

## 4 DISCUSSION

The stepped‐wedge LIVE@Home.Path trial aimed to evaluate the effectiveness of a multicomponent intervention on informal caregiver burden and use of care time in dyads of home‐dwelling people with dementia and their caregivers. We did not find an overall improvement in either primary outcome of caregiver burden or use of care time during the 24‐month trial. Nonetheless, the caregivers perceived the LIVE intervention as supportive, reflected by a positive impression of change regarding each of the single LIVE components. This change was most pronounced for the implementation of the LIVE intervention by a municipal coordinator who tailored the services to the users’ individual needs and demonstrates that this personalized human component is highly valued by the caregivers. This secondary finding may assist clinicians and stakeholders designing future health‐care services to people with dementia and their relatives. As the prevalence will more than double over the next 30 years,^28^ there is impetus to support their caregivers.

Multicomponent interventions are more effective than single component interventions in reducing caregiver burden in dementia.^7^ Yet, it is still unclear which components to include in the most effective intervention. The feasibility study of the LIVE@Home.Path trial suggested that patient participation in the decision‐making process is crucial for tailoring care and support.^16^ Therefore, we designed the LIVE intervention in close collaboration with our user representative (R.S.), and selected components already recommended by the stakeholders in the Norwegian Dementia Plan 2020.^6^ Nonetheless, the findings on how caregivers perceived the CGIC revealed that they attributed the most beneficial change to the presence of a municipal coordinator, suggesting that personalized dementia care might be even more important than the various components constituting the intervention.

In the feasibility study of the LIVE@Home.Path, Fæø et al conducted in‐depth focus group interviews with caregivers, revealing that the coordinator filled three major functions: a safety net, a pathfinder and a source of emotional care and support.^29^ This reflects the unmet needs caregivers face in the home‐dwelling setting. The results regarding the coordinator from both the feasibility study and the LIVE@Home.Path trial align with previous work. A meta‐analysis demonstrated that coordinating interventions in dementia care had a positive impact on caregiver burden,^30^ while a systematic review showed that case management in community aged‐care improved caregiver psychological health, well‐being, and unmet service needs.^31^ Therefore, we suggest that future studies should evaluate the cost effectiveness of the implementation of a skilled coordinator tailoring services in municipal dementia care.

There are several probable reasons for the lack of an intervention effect on the primary outcomes in this pragmatic trial. First, the inclusion criteria for caregiver participation were only minimum 1 hour/week with regular contact with the person with dementia and the time spent on caregiving tasks and caregiver burden at baseline were not particularly high. This might imply that a further reduction at group level would be difficult to achieve with our intervention. For instance, the caregivers in the LIVE@Home.Path trial had a lower level of burden compared to the caregivers in the REACH‐II trial.^8^ This also applies to our finding of no intervention effect on the secondary outcomes caregiver's quality of life and symptoms of depression, which at baseline were both in the normal range. Second, the intervention aimed at increasing caregivers’ literacy concerning dementia; consequently, they might become more aware of their role and tasks in providing informal care over the trial. As such, the amount of time they *report* spending on caregiver tasks may increase during and after the intervention, in line with our findings of a non‐statistically significant increase in RUD‐PADL of 11.7 hours/months in the intervention period. If this finding represents a reporting bias rather than a true increase in time spent on caregiving tasks, it may mask a possible intervention effect. Third, we were not able to standardize the usual care provided in the different municipalities due to the pragmatic nature of the study. For instance, many municipalities already offer dementia courses, use of assistive technology, and volunteer services. Even though the aim of the LIVE intervention was to map out and customize the available services to the dyads needs, some dyads could have benefitted from the LIVE components already available in the municipalities before enrollment. This would make it difficult to demonstrate an additional intervention effect during the trial.

LIVE@Home.Path is one of many clinical trials affected by the SARS CoV‐2 pandemic.^32^ During the strictest lockdown, the coordinators successfully provided follow‐up to the dyads by phone, while there was a paucity of the available services constituting the LIVE intervention, such as dementia courses, installation of devices at home, and volunteer services.^12^ Yet, we did not find evidence for a delayed intervention effect in the time‐dependent models, arguing against that the pandemic contributed to the lack of an intervention effect. Nonetheless, we suggest that the pandemic has reframed formal care provision to vulnerable people with dementia at home, and that the lessons learned will inform us on how to optimize remote health‐care services in the future.

To our best knowledge, LIVE@Home.Path is the first trial to examine the effect of an intervention on dyads of home‐dwelling people with dementia and their caregivers using a stepped‐wedge design. We chose this design to provide all participants with the intervention, and to reduce the burden of serving many dyads simultaneously for the coordinators who delivered the intervention. Other strengths of our study include a 24‐month trial with a minimum 6‐month follow‐up period after the intervention and that we succeeded in continuing the trial after the SARS CoV‐2 pandemic outbreak. A high number of dyads were included from multiple sites and levels of health‐care services in Norway, yet the recruitment of White Norwegian dyads solely from health‐care services may limit the generalizability of our findings to informal caregivers to people with dementia already receiving formal care.

Our study has limitations. First, the original power calculation only focused on one outcome and the calculation is considered conservative by not addressing the staggered intervention delivery. Therefore, we conducted additional power calculations by treating the study as an individually randomized stepped‐wedge trial after the protocol had been formalized, and we performed this calculation for both primary outcomes separately. These additional calculations, however, suggest that our original sample size remains sufficient. Second, although we included two primary outcomes due to their relevance to our study, we have not accounted for multiple testing and instead considered the analysis of each primary outcome to address a separate hypothesis. Accounting for multiplicity would reduce the type I error rate spent on each outcome and generally inflate the required sample size. Third, despite the stepped‐wedge design, we had a higher dropout rate than expected, especially after the pandemic outbreak. This may be explained by factors independent of the trial, in particular the availability of nursing home places, which increased due to the opening of new nursing homes in one of the included municipalities. Moreover, when the persons with dementia were admitted to nursing home, we considered the dyad as dropped out, and the level of burden and care time was no longer assessed among the caregivers. This hinders us from exploring how the caregiver burden and informal care time changes when formal care substantially increases, and we suggest that this should be explored in future studies. Fourth, while our analysis includes as many baseline covariates as possible to address outcomes missing at random, it might be possible that the drop‐out mechanism depends on unmeasured factors that can affect the primary outcomes. Such missing not at random mechanisms may introduce bias to the estimation of intervention effects. Finally, we acknowledge that caregivers providing a high amount of care time could experience distinct challenges compared to those with fewer hours. However, stratifying the population into subgroups based on care hours in our trial does not provide sufficient power to investigate this important issue. This should, however, be addressed in future studies.

In conclusion, the LIVE intervention did not reduce caregiver burden nor time spent on caregiving tasks, yet the caregivers to persons with dementia residing at home reported a positive impression of change over the trial. This change was most pronounced for the implementation of the LIVE intervention by trained municipal coordinators who tailored the services to the user's individual needs. This finding should be considered when designing future health‐care services to families affected by dementia.

## CONFLICT OF INTEREST STATEMENT

Dr. Ballard reports personal fees from Johnson and Johnson, Novo Nordisk, ReMynd, Acadia, AARP, Addex, Eli Lilly, GW Pharma, BMS, Janssen, Orion, Sunovion, Suven, Roche, Biogen, and TauRx. Dr. Selbæk reports honoraria for lectures at symposia sponsored by Eisai and Eli‐Lilly and has participated on advisory boards for Eli‐Lilly, Eisai, and Roche regarding disease‐modifying treatment of Alzheimer's disease. Dr. Aarsland reports grants from Roche Diagnostics, consulting fees from Roche Diagnostics, Eisai, Eli Lilly, and GSK. All other authors have no interests to disclose. Author disclosures are available in the supporting information.

## CONSENT STATEMENT

Prior to inclusion, spoken and written informed consent was obtained in direct conversation with all participants in the trial, if capable of providing consent for participation. If not, the informal caregiver or legal advocate provided consent based on their determination on whether the person with dementia would have agreed to participate when he or she was able to make this decision.

## Supporting information



Supporting Information

Supporting Information

Supporting Information

Supporting Information

Supporting Information
