# Supplementary material for: Burden and care time for dementia caregivers in the LIVE@Home.Path trial
Source: Alzheimers Dement. 2025 Mar 5;21(3):e14622. doi: 10.1002/alz.14622 (PMC11881633; doi:10.1002/alz.14622)
Supplement: Supplementary file 1 — Supporting Information [file ALZ-21-e14622-s004.docx]

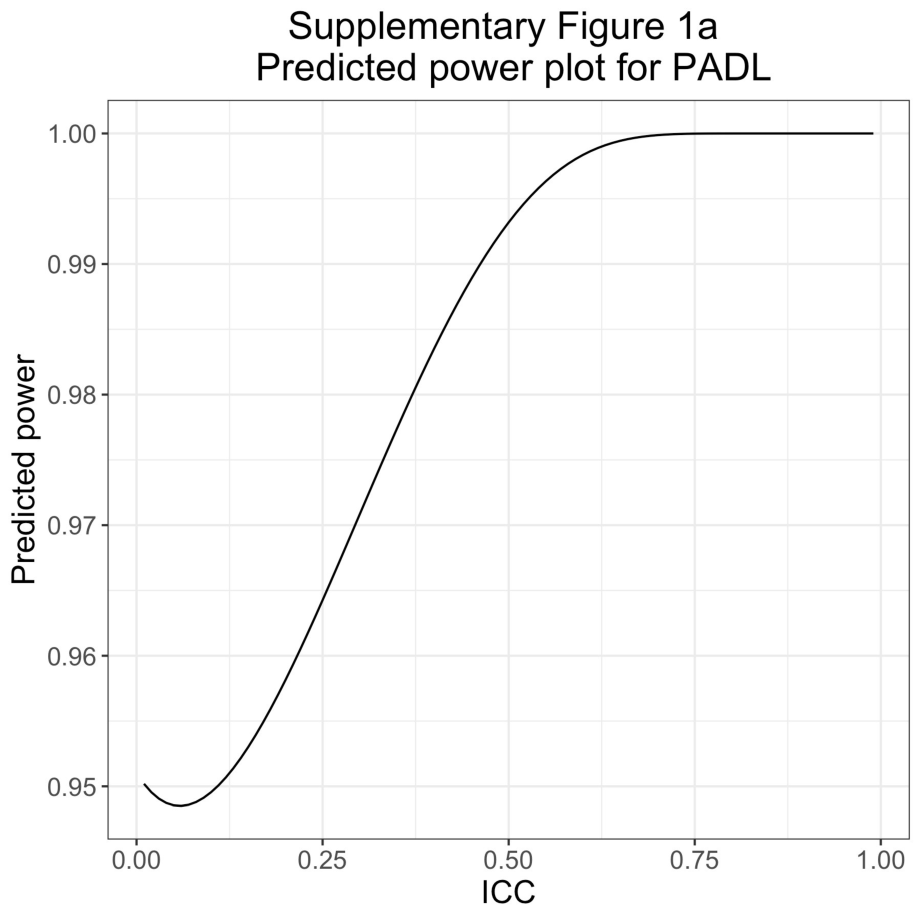


**Supplementary Figure 1a**. Predicted power line for RUD-PADL as a function of ICC in (0, 1), with the effect size of 7h/week, outcome standard deviation of 20, and within-individual correlation of 0.1.
