# Supplementary material for: Burden and care time for dementia caregivers in the LIVE@Home.Path trial
Source: Alzheimers Dement. 2025 Mar 5;21(3):e14622. doi: 10.1002/alz.14622 (PMC11881633; doi:10.1002/alz.14622)
Supplement: Supplementary file 2 — Supporting Information [file ALZ-21-e14622-s003.docx]

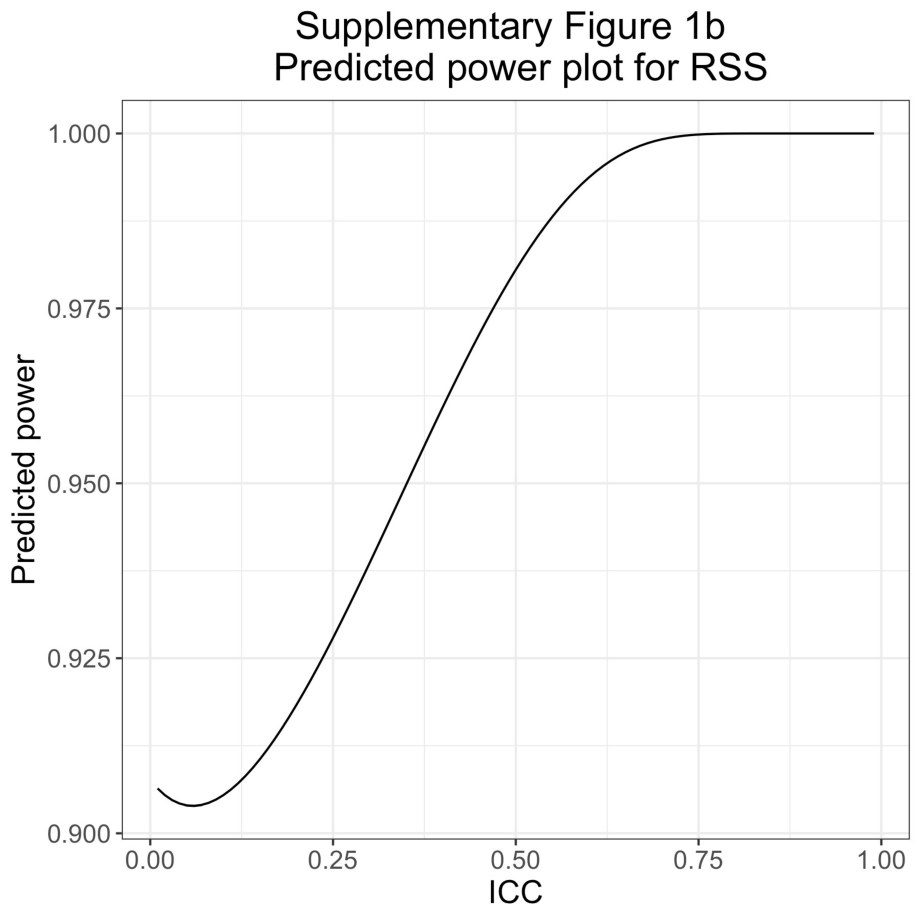


**Supplementary Figure 1b**. Predicted power line for RSS as a function of ICC in (0, 1), with the effect size of 3.5 units, outcome standard deviation of 11, and within-individual correlation of 0.5.
