# Supplementary material for: Burden and care time for dementia caregivers in the LIVE@Home.Path trial
Source: Alzheimers Dement. 2025 Mar 5;21(3):e14622. doi: 10.1002/alz.14622 (PMC11881633; doi:10.1002/alz.14622)
Supplement: Supplementary file 3 — Supporting Information [file ALZ-21-e14622-s002.docx]

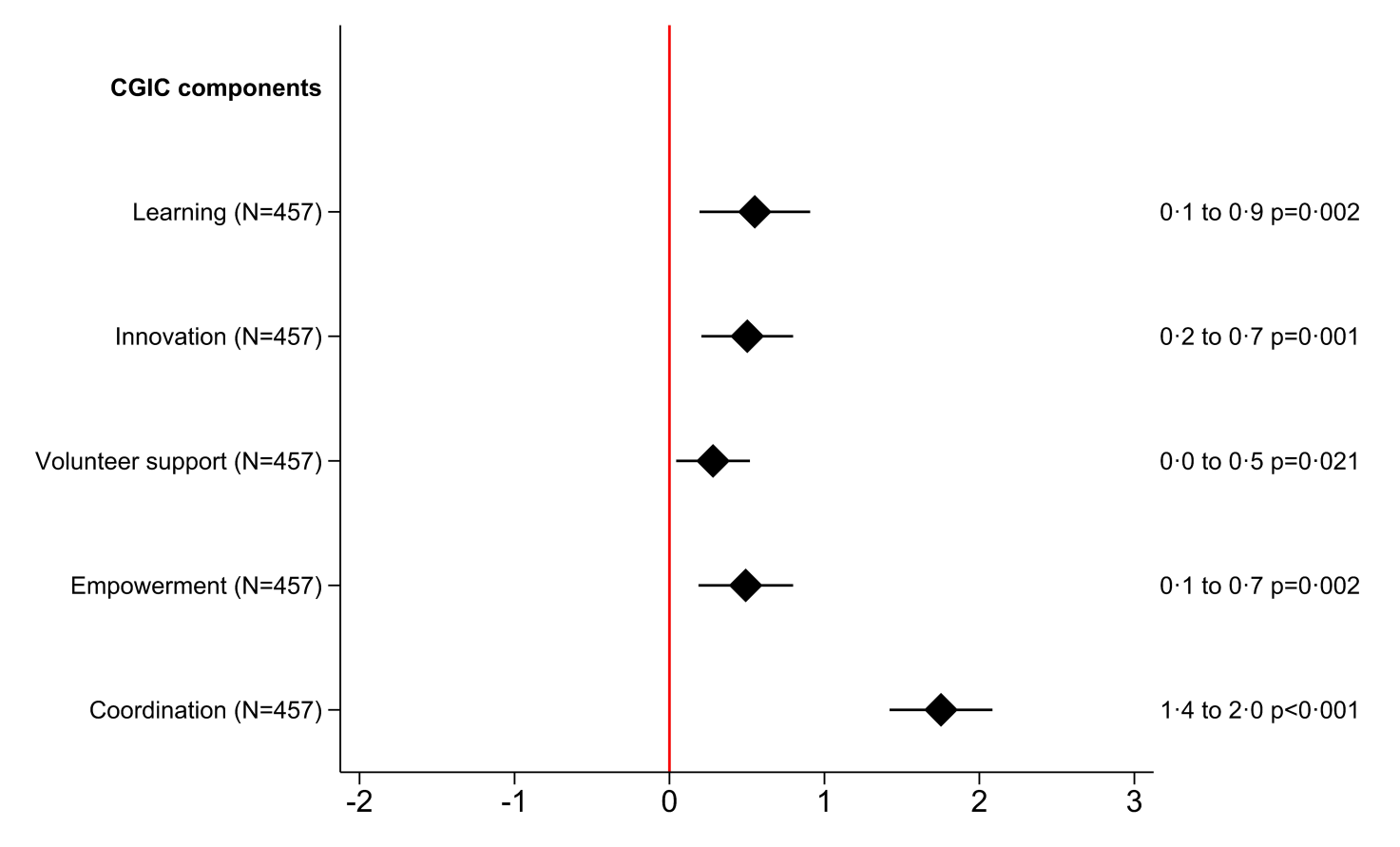


**Supplementary Figure 2:** Mixed effect regression with time-invariant intervention effect – CGIC

The estimates show the effect of LIVE intervention on the four LIVE components and the coordinator evaluated with CGIC. Models are adjusted for baseline covariates: sexes of the person with dementia and caregiver, caregiver age, relation to person with dementia, dementia etiology, presence of pain disorders and personal activities of daily living (total score). N represents the number of observations analyzed in the regression model.
