# Supplementary material for: Burden and care time for dementia caregivers in the LIVE@Home.Path trial
Source: Alzheimers Dement. 2025 Mar 5;21(3):e14622. doi: 10.1002/alz.14622 (PMC11881633; doi:10.1002/alz.14622)
Supplement: Supplementary file 4 — Supporting Information [file ALZ-21-e14622-s005.docx]

Supplementary materials

**Supplementary Table 1:** Model specifications time-dependent intervention effects

|  | *p*=1 | *p*=2 | *p*=3 | *p*=4 |
| --- | --- | --- | --- | --- |
| *s*=3 |  |  | $\delta_{0}^{*}$ | $\delta_{1}^{*}$ |
| *s*=2 |  | $\delta_{0}^{*}$ | $\delta_{1}^{*}$ | $\delta_{1}^{*}$ |
| *s*=1 | $\delta_{0}$ | $\delta_{1}^{*}$ | $\delta_{1}^{*}$ | $\delta_{1}^{*}$ |

The deltas $(\delta)$ constitute the intervention effect, and their subscript defines heterogenous effects where $\delta_{0}$ is the pre-lockdown intervention effect, $\delta_{0}^{*}$is the post-lockdown intervention effect, and $\delta_{1}^{*}$is the follow-up post-lockdown intervention effect. *s:* intervention sequences, *p:* periods.

**Supplementary Table 2:** Within-individual correlation coefficients

|  | *ICC (Time-invariant effect model)* | *ICC (Time-dependent effect model)* |
| --- | --- | --- |
| *Primary outcomes* |  |  |
| RSS | 0.563 | 0.528 |
| RUD-PADL | 0.028 | 0.028 |
| *Secondary outcomes* |  |  |
| RUD-IADL | 0.183 | - |
| RUD-Supervision | 0.081 | - |
| CGIC | 0.209 | - |
| EQ-5D-5L | 0.196 | - |
| GDS | 0.661 | - |

The within-individual correlation estimated from all models based on recommendations from the CONSORT statement. (26)
